# Early myocardial injury in children on doxorubicin for cancer chemotherapy: a cross-sectional study in a tertiary referral centre in Kenya

**DOI:** 10.1186/s12872-024-03922-y

**Published:** 2024-05-20

**Authors:** Nyambura Kariuki, Esther Kimani, Christine Jowi, Dalton Wamalwa, Jacky Y. Suen, John F. Fraser, Nchafatso G. Obonyo

**Affiliations:** 1 Department of Paediatrics and Child Health, School of Medicine, College of Health Sciences, University of Nairobi, KNH, P. O. Box, Nairobi, 19676-00202 Kenya; 2 Initiative to Develop African Research Leaders (IDeAL)/KEMRI-Wellcome Trust Research Programme, P. O. Box 230-80108, Kilifi, Kenya; 3 Kenya Medical Association, Nairobi, Kenya; 4 Critical Care Research Group, The Prince Charles Hospital, 627 Rode Road, Chermside, 4032 Queensland Australia; 5 Institute for Molecular Bioscience, The University of Queensland, 306 Carmody Road, St Lucia, 4067 Queensland Australia

**Keywords:** Paediatric cancers, Chemotherapy, Cardiac toxicity, Echocardiography, Cardiac troponin

## Abstract

**Introduction:**

Use of doxorubicin, an anthracycline chemotherapeutic agent has been associated with late-occurring cardiac toxicities. Detection of early-occurring cardiac effects of cancer chemotherapy is essential to prevent occurrence of adverse events including toxicity, myocardial dysfunction, and death.

**Objective:**

To investigate the prevalence of elevated cardiac troponin T (cTnT) and associated factors of myocardial injury in children on doxorubicin cancer chemotherapy.

**Methods:**

*Design:* A cross-sectional study.

*Setting and subjects:* A hospital-based study conducted on children aged 1-month to 12.4-years who had a diagnosis of cancer and were admitted at Kenyatta National Hospital (KNH).

*Interventions and outcomes:* The patients underwent Echocardiography (ECHO) before their scheduled chemotherapy infusion. Twenty-four (24) hours after the chemotherapy infusion the patients had an evaluation of the serum cardiac troponin T (cTnT) and a repeat ECHO. Myocardial injury was defined as cTnT level > 0.014 ng/ml or a Fractional Shortening (FS) of < 29% on ECHO.

**Results:**

One hundred (100) children were included in the final analysis. Thirty-two percent (32%) of the study population had an elevated cTnT. A cumulative doxorubicin dose of > 175 mg/m^2^ was significantly associated with and elevated cTnT (OR, 10.76; 95% CI, 1.18–97.92; *p* = 0.035). Diagnosis of nephroblastoma was also associated with an elevated cTnT (OR, 3.0; 95% CI, 1.23–7.26) but not statistically significant (*p* = 0.105). Nine percent (9%) of the participants had echocardiographic evidence of myocardial injury.

**Conclusion:**

When compared to echocardiography, elevated levels of cTnT showed a higher association with early-occurring chemotherapy-induced myocardial injury among children on cancer treatment at a tertiary teaching and referral hospital in Kenya.

**Supplementary Information:**

The online version contains supplementary material available at 10.1186/s12872-024-03922-y.

## Introduction

The mainstay of management of most paediatric malignancies is chemotherapy which has significantly increased the survival of most children with malignancies from 20–30% in 1960s when chemotherapy was introduced [1], to 83% in 2014 [2]. However, the use of some of these chemotherapeutic agents such as anthracyclines is limited by their toxicity profiles especially cardiac toxicity. Doxorubicin is an anthracycline compound that was isolated from the pigment-producing bacterium *Streptomyces peucetius var. caesius* in the early 1960s [3]. Its chemotherapeutic action is by blocking the topo-isomerase-2 enzyme thus slowing the proliferation of cancerous cells. However, it also causes dilated cardiomyopathy, congestive cardiac failure and even sudden cardiac death in the long term [4]. Up to 65% of paediatric cancer survivors who received chemotherapy with doxorubicin developed late cardiac toxicities [5]. Early detection of doxorubicin-induced cardiac toxicities is essential in preventing the late-occurring cardiac toxicity of this agent [6]. Currently the most common method of detecting doxorubicin-induced cardiac injury is the evaluation of functional parameters such as Fractional Shortening (FS) and Ejection Fraction (EF) on echocardiography [7]. Indeed, these echocardiographic parameters reliably detect late-occurring changes of chemotherapy induced cardiac injury.

Cardiac troponin T (cTnT) has been used as a marker of myocardial injury, especially in adults with acute coronary syndromes. Plasma levels of cTnT rise 4–6 h after myocardial infarction, peaks at 24 h and remains elevated for 10–14 days [8]. Increased plasma cTnT levels have been informative in early detection of chemotherapy-induced myocardial injury in rats [9]. Studies in children on cancer chemotherapy have shown that an increase in cTnT in the first 90-days of treatment is associated with reduced left ventricular (LV) mass and LV end-diastolic posterior wall thickness, 4 years after completion of chemotherapy [10]. The positive predictive value (PPV) and the negative predictive value (NPV) of cardiac troponins in detecting cardiac toxicity of chemotherapy are 84% and 99% respectively [6]. Thus, troponins stratify cardiac risk in the very early phase long before impairment of heart function and symptoms develop. Studies in adult populations have also shown that there is a close relationship between the peak value of cardiac troponins and the degree of late LV EF reduction [11].

Three types of anthracycline-induced cardiotoxicities have been described (acute, early-onset chronic and late-onset chronic), however data supporting this classification are lacking in the literature [12]. The European Society of Cardiology (ESC) 2016 guidelines classify anthracycline-induced cardiac dysfunction (ACD) as either early or late depending on whether the ACD develops within or after the first year of treatment respectively [13].

Some of the factors associated with myocardial injury in children on cancer chemotherapy include cumulative anthracycline dose, younger age, female gender, being of African ancestry and use of other cardiotoxic agents [5, 14, 15].

The aim of this study was to determine the prevalence of early-occurring myocardial injury, as determined by elevated cTnT in African children on cancer chemotherapy and to explore the factors associated with an elevated cTnT.

## Methods

### Study population

The study population was children aged between 1 month and 12.4-years old who had a diagnosis of cancer and who were in the first year of treatment receiving chemotherapy. All the children included had normal oxygen saturation levels > 94%. Children with pre-existing congenital or acquired heart disease including rheumatic heart disease, as determined by echocardiography, were excluded. Additionally, children who had an abnormal renal function were ineligible for cancer chemotherapy and thus not considered for inclusion in the study. These children first underwent treatment to optimise their renal function before being considered for chemotherapy.

### Study setting

The study setting was the Kenyatta National Hospital (KNH), located in Nairobi, the capital city of Kenya. It is the largest tertiary-level national referral hospital and also the main teaching hospital for the University of Nairobi. It serves as the major referral hospital for all paediatric cancer patients in Kenya. Approximately 7–10 children with malignancies are referred to KNH every month.

### Study design and duration

We conducted a 6-month cross-sectional hospital-based study between 1st July and 31st December 2016 in accordance with the STROBE guideline on cross-sectional studies.

### Ethics approval

The study protocol was approved by the Kenyatta National Hospital-University of Nairobi Ethics Review Committee (KNH-UON ERC), approval protocol number P220/03/2016. All parents/legal guardians of the study participants gave written informed consent before participation in the study.

### Study outcome

The outcome of this study was to determine the prevalence of myocardial injury. Myocardial injury was defined as:

Cardiac Troponin T (cTnT) level greater ( >) than 0.014 ng/ml.

Or.

Echocardiographic evidence of left ventricular Fractional Shortening of less ( <) than 29%.

### Data collection

#### Cardiac evaluation

We assessed cardiac function of all study participants using physical examination and echocardiography (ECHO), which was performed by a paediatric cardiologist, in the presence of the study investigators (EK and CJ). The first 2-D ECHO was done prior to the participant receiving their scheduled chemotherapy infusion. We assessed left ventricular function by determining the EF and FS. Diastolic function was measured by peak early velocity (E wave), peak atrial velocity (A wave), E wave/A wave ratio (E/A), E deceleration and isovolumetric relaxation time (IVRT). Patients underwent a second 2-D ECHO 24 h after receiving their scheduled chemotherapy. At the time of performing the second 2-D ECHO, the paediatric cardiologist was blinded to the patients’ cTnT level.

#### Biochemical analysis

All the blood samples for cTnT evaluation were obtained from the antecubital vein. The serum cTnT samples were drawn and measured 24 h after the participants had received their scheduled chemotherapy treatment. The cTnT level was measured using the Elecsys troponin T STAT Immunoassay (Roche Diagnostics). The lower limit of detection of this assay is 0.003 ng/ml. An abnormal value was defined as any detectable level of cTnT, that is, any level above 0.014 ng/ml. All above were recorded in data collection sheets.

### Data analysis

Data collected were entered into a patient data sheet and then transcribed to a computer data base, SPSS version 23.0. Descriptive, univariate, and multivariate analyses were done to determine the factors associated with elevated cTnT. Statistical significance was considered at an alpha (α) level of 0.05 and Bonferroni correction for multiple comparisons was applied in the analyses.

## Results

One hundred (100) children were included in the final analysis. Majority of the children were male, 64 (64%) with a male to female ratio of 1:1.8. The median age was 53 months (IQR = 35–84). The commonest malignancy was nephroblastoma in 33 (33%) participants. The median duration of treatment of 3 months (IQR = 1–8.5 months). Seventy seven percent (77%) were exposed to an anthracycline (mainly doxorubicin) with a median cumulative doxorubicin dose of 90.5 mg/m^2^ (range 0–550). Eighty-eight children (88%) were discharged alive after completion of their chemotherapy treatment. Four children (4%) died while undergoing chemotherapy treatment with only one of these being ascertained as due to heart failure from the case record forms. The cause of death for the other three patients could not be ascertained as it was unclear from the reviewed case record forms. Eight children (8%) were still undergoing treatment by the time the 6-months study duration came to an end.

Table 1 summarises the study population characteristics.

**Table 1:** Demographics and baseline characteristics of the study population

Characteristics	Frequency (%)/ Median (IQR)
**Sex**	
• Females	36 (36%)
Age (months)	53 (35–84)
Weight (kg)	15 (12–19)
Weight/height z score (> median score)	67(67%)
**Malignancy group**	
• Leukaemia	29 (29%)
• Lymphoma	14 (14%)
• Solid tumour	57 (57%)
**Malignancy Type**	
• Nephroblastoma	33 (33%)
• Acute Lymphocytic leukaemia	27 (27%)
• Non-Hodgkin lymphoma	10 (10%)
• Rhabdomyosarcoma	9 (9%)
• Hodgkin Lymphoma	4 (4%)
• Neuroblastoma	4 (4%)
• Others	13(13%)
**Malignancy stage (for lymphoma and solid tumours only)**	
• Stage 1	21 (21%)
• Stage 2	6 (6%)
• Stage 3	22(22%)
• Stage 4	21(21%)
Treatment Duration (months)	3 (0.25–8.5)
**Exposed to anthracyclines**	
• Yes	77 (77%)
• No	23(23%)
**Anthracycline cumulative dose category (mg/m**^**2**^**)**	
• < 50	37 (37%)
• 51–100	29 (29%)
• 101–175	17 (17%)
• > 175	17 (17%)
**Exposed to alkylating agents**	
• Yes	61 (61%)
• No	39 (39%)
**Outcomes**	
• Discharged alive	88 (88%)
• Mortality	4 (4%)
• Still on treatment at the end of 6-month follow-up	8 (8%)

**Fig. 1:**
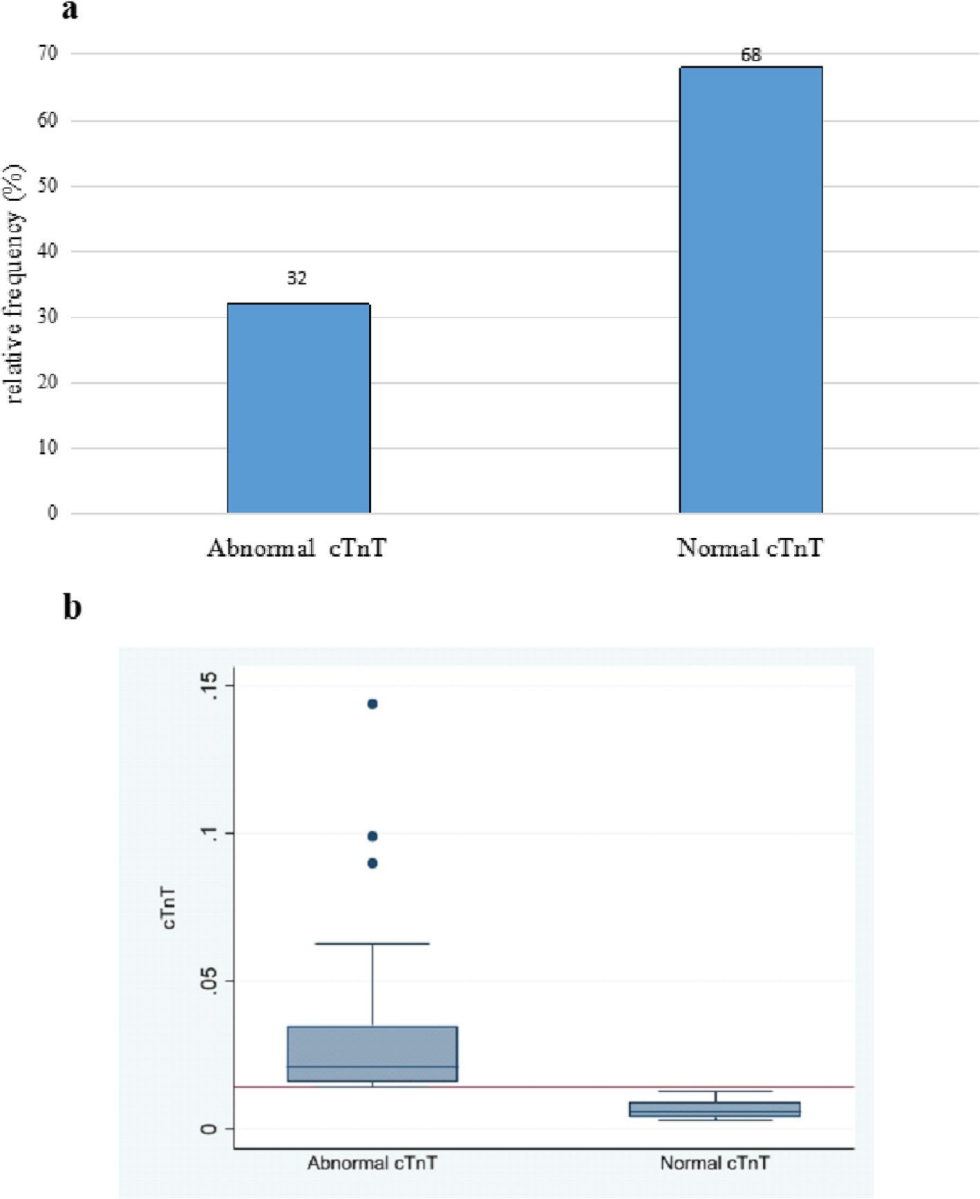
**a** Bar Graph showing the proportion of participants with abnormal *vs* normal cardiac troponin T (cTnT) levels. **b** Box plot showing the absolute cardiac troponin T (cTnT) levels in the elevated and normal groups

The proportion of participants with elevated cTnT levels was, 32% (95% CI, 23.67%—41.66%), as demonstrated in Fig. 1(a) and 1(b).

Participants with a diagnosis of nephroblastoma had a threefold significant increase in the odds of having an elevated cTnT compared to those with other malignancy types, (OR, 3; 95% CI, 1.23–7.26; *p* = 0.015). Participants who received a cumulative dose of more than 175 mg/m^2^ of doxorubicin had a more than tenfold increase in the odds of having an elevated cTnT level, (OR, 10.76; 95% CI, 1.182–97.92; *p* = 0.035). The summary of the univariate and logistic regression models is as shown in Tables 2 and 3, respectively. The median (IQR) doxorubicin dose by age-distribution is presented in Supplemental Table S1.

**Table 2:** Summary of univariate analysis of factors associated with elevated cardiac troponin T (cTnT)

**Characteristics**	**Elevated cTnT** **n (%)** ***n* = 32**	**Normal cTnT n (%)** ***n* = 68**	**OR (95% CI)**	***P*-value**
Median Age (months.)	44 (22–80)	60 (37–89)	0.99 (0.97–1.003)	0.08
**Gender**				
Female (IQR)	15 (46.9)	21(30.9)	2.098 (0.877– 5.020)	0.12
Males	17 (53.1)	47 (69.1)		
WHO W/H Z score (< -3)	2 (6.5)	6 (9.2)	1.037 (0.404–2.660)	0.90
**Malignancy group**				
Solid tumour	23(71.9)	34 (50)		**0.039**
Leukaemia	3 (9.4)	26 (38.2)	-	**0.012**
Lymphoma	6 (18.8)	8 (11.8)		
**Malignancy type**				
Nephroblastoma	16(50)	17(25)		**0.013**
ALL	3(9.4)	24(35.3)		
NHL	5(15.6)	5(7.4)	-	
Rhabdomyosarcoma	2(6.2)	7(10.3)		
others				
**Malignancy stage**				
Stage 1	8(29.6)	13 (30.2)	-	-
Stage 2	1(3.7)	5 (11.6)	2.69(0.26–27.80)	0.40
Stage 3	10 (37)	12 (27.9)	4.17(0.41–41.78)	0.22
Stage 4	8(29.6)	13(30.2)	3.077(0.30–31.33)	0.64
**Exposure to anthracyclines**				
Yes	25 (78.1)	52(76.5)	0.94(0.34–2.60)	0.92
No	7(21.9)	16(23.5)		
**Cumulative anthracycline dose (mg/m2)**				
< 50	10 (31.2)	27(39.5)	-	
50–100	11(34.4)	18 (26.5)	1.65(0.58–4.68)	0.34
100–175	3(9.4)	14(20.6)	0.58(0.13–2.44)	0.45
> 175	8(25)	9(13.2)	2.4(0.72–7.96)	0.15
**Exposure to alkylating agents**				
Yes	22 (68.8)	39 (57.4)	0.64 (0.26–1.56)	0.28
No	10 (31.2)	29 (42.6)

**Table 3:** Logistic regression of factors associated with elevated cardiac troponin T (cTnT)

Outcome: cTnT (Elevated vs. Normal)	Odds ratio (95% CI)	*P*-value
Malignancy type		
Nephroblastoma	3 (1.23–7.26)	**0.015**
Cumulative Adriamycin Dose (mg/m2)		
> 175	10.76(1.18–97.92)	**0.035**

Nine (9%) study participants exhibited abnormal Fractional Shortening, FS (95%CI: 4.38%—15.9%) and 5 (5%) had an abnormal Ejection Fraction, EF (95% CI: 1.67%—10.44%). There was no significant correlation between the EF and the level of cTnT (*R* = 0.095, SE 0.31 and *p* = 1.00). The baseline and post-treatment echo data are presented in Supplemental Table S2.

## Discussion

We conducted a cross-sectional study investigating the prevalence of and factors associated with early-occurring myocardial injury among children within the first year of cancer diagnosis and receiving cancer chemotherapy at KNH. The main findings of this study can be summarised as follows: (a) A third of the patients studied had elevated levels of cTnT with less than 10% showing abnormalities on echocardiography; (b) nephroblastoma was the commonest malignancy seen in a third of the study patients; (c) more than three-quarters of the study patients received anthracycline-based chemotherapy (mainly doxorubicin) which was associated with elevated cTnT levels at higher doses.

Anthracycline-induced cardiotoxicity has previously been reported to carry a poor prognosis with approximately 50% mortality occurring in the first year of developing congestive cardiac failure [16]. In 1979, Chlebowski reviewed the anti-neoplastic use of doxorubicin hydrochloride noting it was effective in a wide range of malignancies but its long-term use was limited by a dose-dependent cardiomyopathy [17]. Over 60% of children with cancers are presently on treatment with anthracyclines [18]. Acute cardiotoxicity secondary to anthracycline therapy can manifest within 2–3 days of administration [19] with a reported incidence of approximately 11% [20, 21]. However, a much lower incidence of 1.7% has been reported for anthracycline-induced chronic cardiotoxicity [16] which may manifest from within 30-days until 10-years since administration of the last dose [19]. The lower incidence of chronic cardiotoxicity notwithstanding, anthracycline anti-neoplastic therapy in childhood and adolescence have been shown to be a predisposing factor for development of adulthood cardiomyopathy [22]. Other reported cardiac effects of anthracyclines include congestive heart failure, higher incidence of cardiac transplantation and risk of death [23]. Some of the acute symptoms that have been associated with reversible myocardial oedema induced by anthracyclines include: Sinus tachycardia, paroxysmal non-sustained supraventricular tachycardia or premature atrial and ventricular beats that may all manifest as palpitations with accompanying chest pain [21, 24]. Reported risk factors for the development of anthracycline-induced cardiomyopathy include factors such as extremes of age (i.e., very young and very old people) and presence of a pre-existing or a history of cardiovascular disease [21, 22]. The reported frequency of clinical cardiotoxic effects of anthracyclines among children varies from 0–16% [18]. This study focused on children, whose median age was 53 months, in whom we found twice as high (32%) a frequency of cardiotoxicity (elevated cTnT) compared to those reported in the literature. Generalisability of these findings will be limited to similar patient populations because children with major co-morbidities including congenital and acquired cardiac disease, renal failure or low oxygen saturations were not included in this study.

The exact mechanism(s) of anthracycline-induced cardiotoxicity are not fully understood. These are however, recognised as different from the anti-neoplastic effects of anthracyclines which include intercalation into deoxyribonucleic acid (DNA), inhibition of DNA binding and cross-linking, inhibition of topoisomerase II leading to DNA damage and induction of apoptosis [21, 25, 26]. Proposed mechanisms of cardiotoxicity are increased lipid peroxidation and a higher oxidative stress [18] as seen from reduced levels of antioxidants such as sulfhydryl groups [27], increased levels of reactive oxygen species [28] and intracellular hydrogen peroxidation [29]. Increased formation of superoxides also occurs due to induction of endothelial nitric oxide synthase by anthracyclines [30, 31] and mitochondrial damage in cardiac tissue [19]. Anthracyclines such as doxorubicin have also been shown to have a high affinity for iron leading to formation of complexes that alter iron metabolism and react with oxygen to trigger further production of reactive oxygen species [32]. There are reports of reduced myocardial contractile function with anthracycline therapy. Among childhood cancer survivors, the frequency of late-occurring cardiotoxicity defined in terms of abnormal echocardiography findings has been reported to be as high as 57% [18]. In this study, abnormal echocardiography findings were seen in 9% (reduced fractional shortening) and 5% (reduced ejection fraction). This lower frequency of echocardiographic abnormalities seen in our study compared to reports in literature could be attributed to a shorter treatment duration reported (median 3-months) and relatively smaller sample size. Some of the proposed mechanisms for contractile dysfunction with anthracycline therapy include; downregulation of both the sarcoplasmic reticular ATPase [33] and the contractile actin and myosin proteins [19]; differentiation of cardiac cells to a pro-fibrotic phenotype [34] and induction of cardiomyocyte apoptosis [35]. Studies on cumulative anthracycline dose in clinical settings have shown a relationship between the degree of cardiac dysfunction and troponin levels with significant increases in cTnT after 2–4 cycles of anthracycline chemotherapy [36]. In evaluating anthracycline-induced cardiac injury for paediatric cancer patients, troponins are reliable biomarkers [37] since repeated anthracycline chemotherapy exposure leads to detectable troponin levels in peripheral circulation [38]. This is consistent with findings from our study whereby 77% of patients were exposed to anthracyclines with 32% having elevated levels of cTnT in a median treatment duration of 3-months.

## Limitations

This was a cross-sectional study that included one hundred paediatric patients on chemotherapy treatment at a tertiary teaching and referral hospital in Kenya exploring the diagnostic performance of cardiac troponin T *versus* echocardiography in detecting early-occurring doxorubicin-induced cardiac toxicity. The reduction in echocardiographically assessed ejection fraction and fractional shortening within 24 h of commencing treatment was a notable observation in this cohort of patients, however, the data available do not provide any plausible explanation for this and furthermore follow-up echo data was missing in over a third of patients. Additionally, cardiac troponin was not assessed prior to the start of chemotherapy, thus this analysis can only infer association but not causation. Cardiac strain could not be assessed due to logistical reasons. More robust local evidence is needed including prospective randomised controlled clinical trials encompassing a wider range of the chemotherapeutic agents used for treatment of paediatric cancers to investigate causation and incorporate strain and myocardial work assessment as well. The included patients had normal oxygen saturations no serious co-morbidities including cardiac, renal or other metabolic conditions. Future research should investigate cancer patients with co-morbidities and also explore non-cardiac and pulmonary causes of elevated troponin.

## Conclusion

In this study, early-occurring chemotherapy-induced cardiotoxicity was evaluated among 100 children undergoing treatment for malignancies. Nephroblastoma was the commonest childhood malignancy and anthracyclines were the predominant anti-neoplastic compounds administered. We found that elevated levels of cTnT showed a higher association with early-occurring doxorubicin-induced myocardial injury among children on cancer treatment at a tertiary teaching and referral hospital in Kenya when compared to echocardiographic evidence of reduced cardiac function. We recommend that future research in this field should explore the utility of cTnT as a screening test for detection of early-occurring myocardial injury among cancer patients commencing chemotherapy and follow-up patients for longer duration of time.

### What is already know on this topic

- 1. Introduction of chemotherapy has significantly increased the survival rate of most children with malignancies.
- 2. However, the use of anthracycline chemotherapeutic agents such as doxorubicin has been associated with long-term cardiac effects leading to dilated cardiomyopathy, congestive cardiac failure, and even sudden death. The long-term effects are irreversible hence the need to detect them early and modify treatment regimens early enough. Cardiac troponins enable early detection as opposed to echocardiography which is the standard of care.

### What this study adds

- 1. There is limited data on the prevalence of early-onset myocardial injury in African children undergoing cancer chemotherapy with anthracyclines such as doxorubicin.
- 2. Diagnosis of early-onset myocardial injury and the factors associated with it among African children on doxorubicin cancer chemotherapy has not been previously described in the literature.

Cardiac troponin assays are comparatively cheaper (when batch-processed), and their interpretation is relatively easier (unlike echocardiography which has to be performed and reported by a specialist paediatric sonographer and cardiologist respectively). This allows for primary care physicians to detect early onset cardiac injury without the need for sophisticated echocardiographic equipment. It is also more affordable to the patient.

### Supplementary Information

Supplementary Material 1.

## Data Availability

Analysed data is provided within the manuscript and all raw data is available from the corresponding author on reasonable request.

## Abbreviations

ACD: Anthracycline-induced cardiac dysfunction
CCRG: Critical Care Research Group
cTnT: Cardiac troponin T
DNA: Deoxyribonucleic acid
ECHO: Echocardiography
EF: Ejection fraction
ERC: Ethics Review Committee
ESC: European Society of Cardiology
FS: Fractional shortening
IDeAL: Initiative to Develop African Research Leaders
IMB: Institute of Molecular Bioscience
IQR: Inter-quartile range
IVRT: Isovolumic relaxation time
KEMRI: Kenya Medical Research Institute
KNH: Kenyatta National Hospital
LV: Left ventricle
NPV: Negative predictive value
OR: Odds ratio
PPV: Positive predictive value
SPSS: Statistical Package for Social Sciences
STROBE: Strengthening the reporting of observational studies in epidemiology
TPCH: The Prince Charles Hospital
UoN: The University of Nairobi
UQ: The University of Queensland
