# Supplementary material for: Early myocardial injury in children on doxorubicin for cancer chemotherapy: a cross-sectional study in a tertiary referral centre in Kenya
Source: BMC Cardiovasc Disord. 2024 May 20;24:260. doi: 10.1186/s12872-024-03922-y (PMC11103839; doi:10.1186/s12872-024-03922-y)
Supplement: Supplementary file 1 — Supplementary Material 1. [file 12872_2024_3922_MOESM1_ESM.docx]

**Supplementary Table S1: Doxorubicin dose and cardiac troponin level by age-group**

| **Age-group, definition (n, %)** | **Doxorubicin dose in mg/m^2^, median (IQR)** | **Cardiac troponin T in ng/mL, median (IQR)** |
| --- | --- | --- |
|  |  |  |
| 1. Infants, 28-days to 12 months (3, 3%) | 89 (77-550) | 0.034 (0.014-0.099) |
| 2. Toddlers, 13-months to 2 years (13, 13%) | 0 (0-68) | 0.014 (0.009-0.019) |
| 3. Early childhood, 2-5 years (38, 38%) | 86 (39-100) | 0.007 (0.004-0.012) |
| 4. Middle childhood, 6-11 years (42, 42%) | 100 (40-188) | 0.007 (0.004-0.012) |
| 5. Adolescence, 12-18 years (4, 4%) | 143 (103-234) | 0.021 (0.010-0.028) |

**Supplementary Table S2: Summary of baseline and post-treatment echocardiography by age-group**

| **Age-group, definition** | **Baseline echocardiography** | | | **Post-treatment echocardiography**  **(median treatment duration 3 months; IQR 1-8.5 months)** | | |
| --- | --- | --- | --- | --- | --- | --- |
|  | **n (%)** | **Ejection fraction %, median (IQR)** | **Fractional shortening %, median (IQR)** | **n (%)** | **Ejection fraction %, median (IQR)** | **Fractional shortening %, median (IQR)** |
| 1. Infants, 28-days to 12 months | 2 (2%) | 69 (66-72) | 38 (36-39) | 2 (4%) | 55 (47-62) | 28 (23-32) |
| 2. Toddlers, 13-months to 2 years | 13 (14%) | 62 (60-66) | 31 (31-35) | 5 (10%) | 64 (60-66) | 31 (30-38) |
| 3. Early childhood, 2-5 years | 37 (39%) | 63 (61-67) | 32 (31-36) | 22 (43%) | 64 (60-66) | 33 (30-36) |
| 4. Middle childhood, 6-11 years | 39 (42%) | 65 (62-69) | 34 (32-37) | 19 (37%) | 62 (60-69) | 33 (29-37) |
| 5. Adolescence, 12-18 years | 3 (3%) | 62 (62-69) | 33 (32-37) | 3 (6%) | 70 (68-73) | 40 (35-41) |
